# A new species of *Kerkia* Radoman, 1978 (Caenogastropoda, Hydrobiidae) from Bosnia and Herzegovina

**DOI:** 10.3897/zookeys.973.52788

**Published:** 2020-10-05

**Authors:** Sebastian Hofman, Aleksandra Rysiewska, Artur Osikowski, Andrzej Falniowski

**Affiliations:** 1 Department of Comparative Anatomy, Institute of Zoology and Biomedical Research, Jagiellonian University, Gronostajowa 9, 30-387 Kraków, Poland; 2 Department of Malacology, Institute of Zoology and Biomedical Research, Jagiellonian University, Gronostajowa 9, 30-387 Kraków, Poland; 3 Department of Animal Reproduction, Anatomy and Genomics, University of Agriculture in Krakow, Mickiewicza 24/28, 30-059 Kraków, Poland

**Keywords:** Balkans, cytochrome oxidase, Gastropoda, histone, interstitial, molecular taxonomy, morphology, stygobiont

## Abstract

A new species of *Kerkia*, *K. briani* Rysiewska & Osikowski, **sp. nov.** is described from the spring Polički Studenac Vrelo (Crkvina), adjacent to the Trebišnjica River (Bosnia and Herzegovina) collected with Bou-Rouch technique, pumped from an interstitial habitat 50 cm below the bottom of the spring. The shell, female reproductive organs, and the penis are described and illustrated. Mitochondrial cytochrome oxidase subunit I (COI) and nuclear histone H3 partial sequences confirm the distinctness of the new species, and molecularly based phylogenetic relationships of *Kerkia* are briefly presented.

## Introduction

Mud snails Hydrobiidae are very small or minute snails, whose shells are often approximately 1 mm high. They inhabit surface and subterranean freshwater habitats, although some can also be found in brackish and even marine environments. The family comprises more than 400 extant genera (Kabat and Hershler 1993), many of which are stygobionts. The Balkans, especially their western region, harbours the world’s most diverse stygobiont malacofauna (e.g., Culver and Sket 2000; Culver 2012). The minute dimensions of those snails, coupled with low population densities (e.g., Culver and Pipan 2009, 2014), result in very poor knowledge of their biology, speciation, and taxonomy. A few specimens are sometimes flooded out of the substrate to the surface. Otherwise, extensive pumping of the interstitial habitats, applying the Bou and Rouch technique sometimes result in more numerous living specimens.

Radoman (1978) established the genus *Kerkia* Radoman, 1978, with the type species *Hauffenia kusceri* Bole, 1961, known only from the cave Krška jama in Slovenia. He described morphology and anatomy of those minute snails, clinging to the rocks in the underground section of the sinking river Krka (Radoman 1973, 1978, 1983). Later, another species of the genus, *K. brezicensis* Bodon & Cianfanelli, 1996, was described from a karstic spring at the entry to Dvorce village, southeast of Brežice in Slovenia. *Hauffenia jadertina* Kuščer, 1933 from the source of the river Jadro near Split in Croatia, as well as *H. jadertina sinjana* Kuščer, 1933 from a spring Zužino Vrelo in the Cetina valley also in Croatia, based on their anatomy, were synonymised and transferred to the genus *Kerkia* by Beran et al. (2014), who also described a new species *Kerkia kareli* Beran, Bodon & Cianfanelli, 2014, from an old well near Povljana on island Pag in Croatia. They provided descriptions and illustrations of the shells, protoconchs, radulae, and soft part morphology and anatomy as well for all the three Croatian taxa. Rysiewska et al. (2017) demonstrated molecular distinctness of those species of *Kerkia*.

In September of 2019, in the spring Polički Studenac Vrelo (Crkvina), adjacent to the Trebišnjica River, we found *Emmericia expansilabris* Bourguignat, 1880, *Sadleriana* sp., *Anagastina vidrovani* (Radoman, 1973), and *Ancylus recurvus* Martens, 1873. Pumping of the interstitial fauna from sediments below the spring resulted in the collection of a few most probably stygophilic *Radomaniola*, but also the typically stygobiont *Montenegrospeum* Pešić & Glöer, 2013 and *Kerkia*. The representatives of the latter genus did not belong to any species known so far, and in the present paper we describe this new species and discuss its relationships.

## Materials and methods

**Figure 1.**
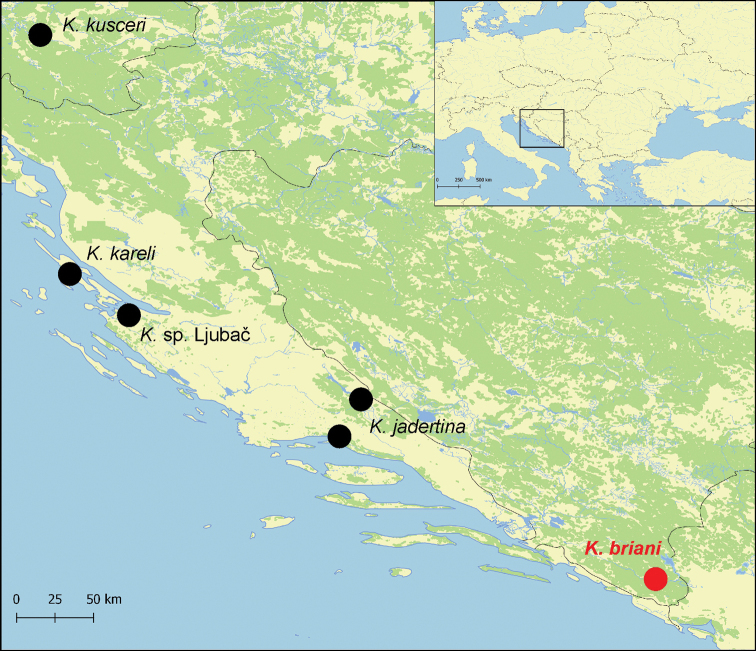
Localities of *Kerkia* used for phylogeny.

**Figure 2.**
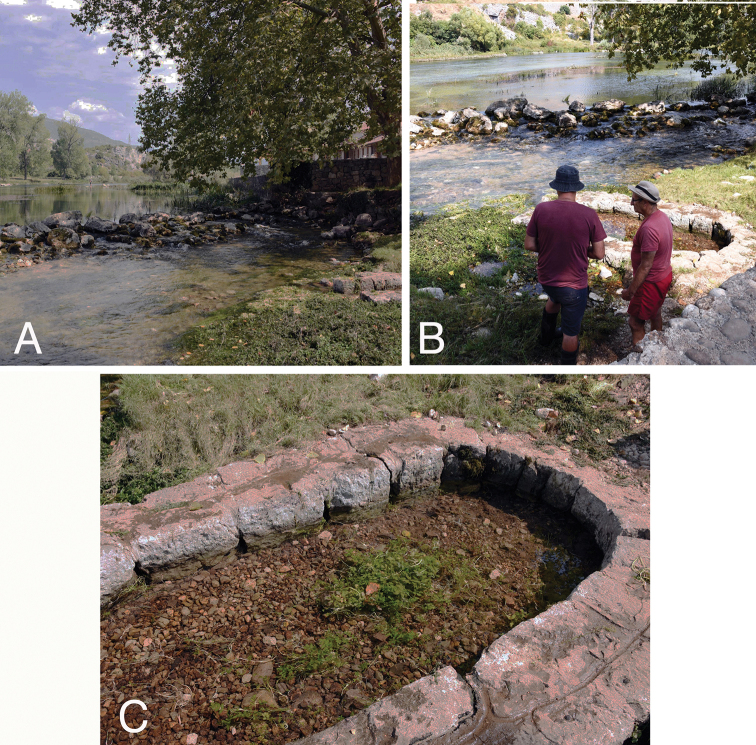
Type locality of *Kerkia briani* sp. nov.: **A, B** River Trebišnjica with the spring Polički Studenac Vrelo at its right bank **C** the spring from where interstitial snails were pumped.

The snails were collected at the spring Polički Studenac Vrelo (Crkvina), adjacent to the Trebišnjica River (42°42'46.4"N, 18°21'54.5"E), near Trebinje, Bosnia and Herzegovina (Fig. 1). The spring, situated at the right bank of the river (Fig. 2A, B) was in the form of a small shallow pool surrounded by a wall made of stones, with a gravel bottom (Fig. 2C). The Bou–Rouch method (Bou and Rouch 1967) was used to sample interstitial fauna below the spring bottom, at the depth of ca. 50 cm. The tube was inserted in the substrate five times, and 20 litres were pumped each time. Samples were sieved through 500 μm sieve and fixed in 80% analytically pure ethanol, replaced two times, and later sorted. Next, the snails were put in fresh 80% analytically pure ethanol and kept in -20 °C temperature in a refrigerator.

**Figure 3.**
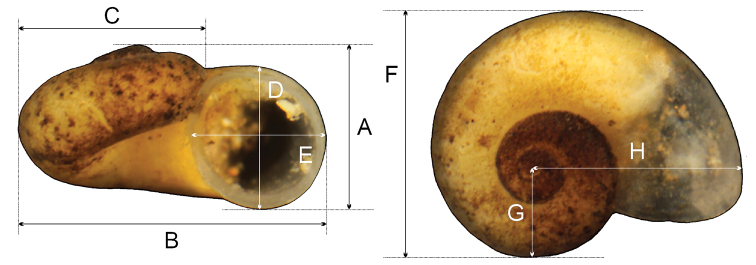
Measurements of the shell.

The shells were photographed with a Canon EOS 50D digital camera, under a Nikon SMZ18 microscope. The dissections were done under a Nikon SMZ18 microscope with dark field, equipped with Nikon DS-5 digital camera, whose captured images were used to draw anatomical structures with a graphic tablet. Measurements of the shell (Fig. 3) were taken using ImageJ image analysis software (Rueden et al. 2017).

Snails for molecular analysis were fixed in 80% ethanol, changed twice, and later stored in 80% ethanol. DNA was extracted from whole specimens; tissues were hydrated in TE buffer (3 × 10 min), total genomic DNA was extracted with the SHERLOCK extraction kit (A&A Biotechnology), and the final product was dissolved in 20 μl of tris-EDTA (TE) buffer. The extracted DNA was stored at -80 °C at the Department of Malacology, Institute of Zoology and Biomedical Research, Jagiellonian University in Kraków (Poland).

DNA coding for mitochondrial cytochrome oxidase subunit I (COI) and nuclear histone 3 (H3) were sequenced. Details of PCR conditions, primers used, and sequencing are given in Szarowska et al. (2016b). Sequences were initially aligned in the MUSCLE (Edgar 2004) programme in MEGA 6 (Tamura et al. 2013) and then checked in BIOEDIT 7.1.3.0 (Hall 1999). Uncorrected p-distances were calculated in MEGA 6. The estimation of the proportion of invariant sites and the saturation test (Xia 2000; Xia et al. 2003) were performed using DAMBE (Xia 2013). In the phylogenetic analysis additional sequences from GenBank were used as reference (Table 1). The data were analysed using approaches based on Bayesian Inference (BI) and Maximum Likelihood (ML). We applied the GTR model whose parameters were estimated by RaxML (Stamatakis 2014). The Bayesian analyses were run using MrBayes v. 3.2.3 (Ronquist et al. 2012) with defaults of most priors. Two simultaneous analyses were performed, each with 10,000,000 generations, with one cold chain and three heated chains, starting from random trees and sampling the trees every 1,000 generations. The first 25% of the trees were discarded as burn-in. The analyses were summarised as a 50% majority-rule tree. The Maximum Likelihood analysis was conducted in RAxML v. 8.2.12 (Stamatakis 2014) using the ‘RAxML-HPC v.8 on XSEDE (8.2.12)’ tool via the CIPRES Science Gateway (Miller et al. 2010). Two species delimitation methods were performed: Poisson Tree Processes (PTP) (Zhang et al. 2013) and Automatic Barcode Gap Discovery (ABGD). The PTP approach was run using the web server https://species.h-its.org/ptp/, with 100 000 MCMC generations, 100 thinning and 0.1 burn-in. We used RAxML output phylogenetic tree. The ABGD approach using the web server (http://www.abi.snv.jussieu.fr/public/abgd/abgdweb.html) and the default parameters.

**Table 1.** Taxa used for phylogenetic analyses with their GenBank accession numbers and references.

Species	COI/H3 GB numbers	References
*Agrafia wiktori* Szarowska & Falniowski, 2011	JF906762/MG543158	Szarowska and Falniowski 2011/Grego et al. 2017)
*Alzoniella finalina* Giusti & Bodon, 1984	AF367650	Wilke et al. 2001
*Amnicola limosus* (Say, 1817)	AF213348	Wilke et al. 2000b
*Anagastina zetavalis* (Radoman, 1973)	EF070616	Szarowska 2006
*Avenionia brevis berenguieri* (Draparnaud, 1805)	AF367638	Wilke et al. 2001
*Belgrandia thermalis* (Linnaeus, 1767)	AF367648	Wilke et al. 2001
*Belgrandiella kuesteri* (Boeters, 1970)	MG551325/MG551366	Osikowski et al. 2018
*Bithynia tentaculata* (Linnaeus, 1758)	AF367643	Wilke et al. 2001
*Bythinella cretensis* Schütt, 1980	KT353689	Szarowska et al. 2016a
*Bythinella hansboetersi* Glöer & Pešić, 2006	KT381101	Osikowski et al. 2015
*Bythiospeum acicula* (Hartmann, 1821)	KU341350/ MK609536	Richling et al. 2016/Falniowski et al. 2019
*Bythiospeum alzense* Boeters, 2001	KU341355	Richling et al. 2016
*Ecrobia maritima* (Milaschewitsch, 1916)	KX355835/MG551322	Osikowski et al. 2016/Grego et al. 2017
*Daphniola louisi* Falniowski & Szarowska, 2000	KM887915	Szarowska et al. 2014c
*Dalmatinella fluviatilis* Radoman, 1973	KC344541	Falniowski and Szarowska 2013
*Emmericia expansilabris* Bourguignat, 1880	KC810060	Szarowska and Falniowski 2013a
*Erhaia jianouensis* (Y.-Y. Liu & W.-Z. Zhang, 1979)	AF367652	Wilke et al. 2001
*Fissuria boui* Boeters, 1981	AF367654	Wilke et al. 2001
*Graecoarganiella parnassiana* Falniowski & Szarowska, 2011	JN202352	Falniowski and Szarowska 2011
*Graziana alpestris* (Frauenfeld, 1863)	AF367641	Wilke et al. 2001
*Grossuana angeltsekovi* Glöer & Georgiev, 2009	KU201090	Falniowski et al. 2016
*Hauffenia michleri* (Kuščer, 1932)	KT236156/KY087878	Falniowski and Szarowska 2015/ Rysiewska et al. 2017
*Heleobia maltzani* (Westerlund, 1886)	KM213723/ MK609534	Szarowska et al. 2014b/ Falniowski et al. 2019
*Horatia klecakiana* Bourguignat 1887	KJ159128	Szarowska and Falniowski 2014
*Hydrobia acuta* (Draparnaud, 1805)	AF278808	Wilke et al. 2000a
Iglica cf. gracilis (Clessin, 1882)	MH720985/ MH721003	Hofman et al. 2018
*Iglica hellenica* Falniowski & Sarbu, 2015	KT825581/MH721007	Falniowski and Sarbu 2015/Hofman et al. 2018
*Islamia zermanica* (Radoman, 1973)	KU662362/MG551320	Beran et al. 2016/Grego et al. 2017
*Kerkia jadertina* (Kuščer, 1933)	KY087868/KY087885	Rysiewska et al. 2017
*Kerkia jadertina sinjana* (Kuščer, 1933)	KY087873-74/ KY087890-91	Rysiewska et al. 2017
*Kerkia kareli* Beran, Bodon & Cianfanelli, 2014	KY087875-77/ KY087892-94	Rysiewska et al. 2017
*Kerkia kusceri* (Bole, 1961)	KY087867/KY087884	Rysiewska et al. 2017
*Kerkia* sp. Ljubač	KY087872/KY087889	Rysiewska et al. 2017
*Littorina littorea* (Linnaeus, 1758)	KF644330/KP113574	Layton et al. 2014/Neretina 2014, unpublished
*Littorina plena* Gould, 1849	KF643257	Layton et al. 2014
*Lithoglyphus prasinus* (Küster, 1852)	JX073651	Falniowski and Szarowska 2012
*Marstoniopsis insubrica* (Küster, 1853)	AF322408	Falniowski and Wilke 2001
Moitessieria cf. puteana Coutagne, 1883	AF367635/MH721012	Wilke et al. 2001/ Hofman et al. 2018
*Montenegrospeum bogici* (Pešić & Glöer, 2012)	KM875510/MG880218	Falniowski et al. 2014/Grego et al. 2018
*Paladilhiopsis grobbeni* Kuščer, 1928	MH720991/MH721014	Hofman et al. 2018
*Peringia ulvae* (Pennant, 1777)	AF118302	Wilke and Davis 2000
*Pomatiopsis lapidaria* (Say, 1817)	AF367636	Wilke et al. 2001
*Pontobelgrandiella* sp. Radoman, 1978	KU497024/MG551321	Rysiewska et al. 2016/Grego et al. 2017
*Pseudamnicola chia* (E. von Martens, 1889)	KT710656	Szarowska et al. 2016b
*Pseudorientalia* Radoman, 1973 – Lesvos	KJ920490	Szarowska et al. 2014a
*Radomaniola curta* (Küster, 1853)	KC011814	Falniowski et al. 2012
*Sadleriana fluminensis* (Küster, 1853)	KF193067	Szarowska and Falniowski 2013b
*Sadleriana robici* (Clessin, 1890)	KF193071	Szarowska and Falniowski 2013b
*Salenthydrobia ferrerii* Wilke, 2003	AF449213	Wilke 2003
*Sarajana apfelbecki* (Brancsik, 1888)	MN031432	Hofman et al. 2019
*Tanousia zrmanjae* (Brusina, 1866)	KU041812	Beran et al. 2015
*Tricula* sp. Benson, 1843	AF253071	Davis et al. 1998

## Results

### Systematic part

#### Family Hydrobiidae Stimpson, 1865

##### Subfamily Sadlerianinae Szarowska, 2006

**Genus *Kerkia* Radoman, 1978**

###### Kerkia briani

Taxon classificationAnimaliaLittorinimorphaHydrobiidae

Rysiewska & Osikowski sp. nov.

AC9D969E-0270-5EF6-B518-2949909E4588

http://zoobank.org/1F772BD0-3172-42E7-B559-EEA20773BCF1

**Figure 4.**
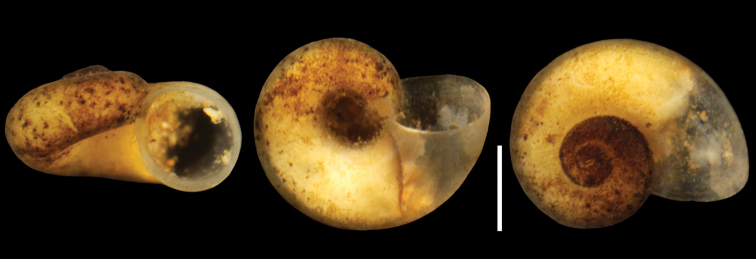
Holotype of *Kerkia briani*. Scale bar: 0.5 mm.

**Figure 5.**
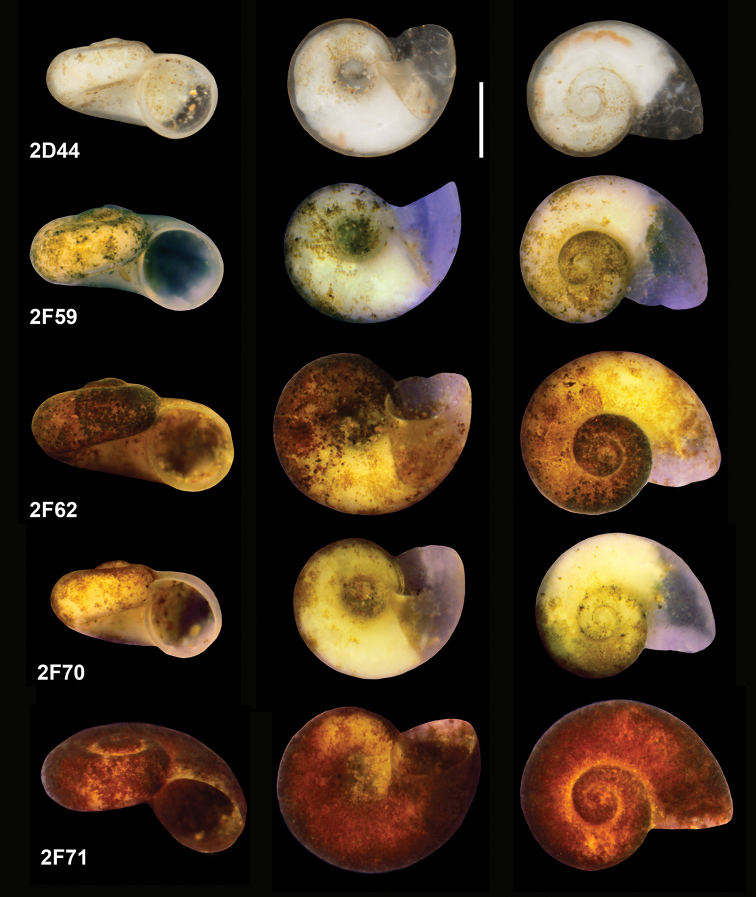
Shell variability of *Kerkia briani*, labels the same as in the molecular trees. Scale bar: 0.5 mm.

**Figure 6.**
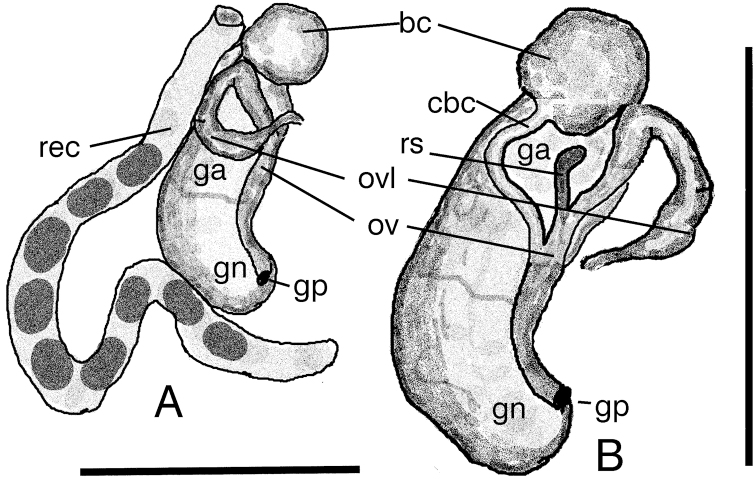
Renal and pallial section of the female reproductive organs of *Kerkia briani*: **A** the loop of oviduct in its normal position and the loop of the rectum **B** the loop of oviduct moved to show the receptaculum seminis and duct of bursa. Abbreviations: bc – bursa copulatrix, cbc – duct of bursa, ga – albuminoid gland, gn – nidamental gland, gp – gonoporus, ov – oviduct, ovl – loop of renal oviduct, rec – rectum, rs – receptaculum seminis. Scale bars: 1 mm.

**Figure 7.**
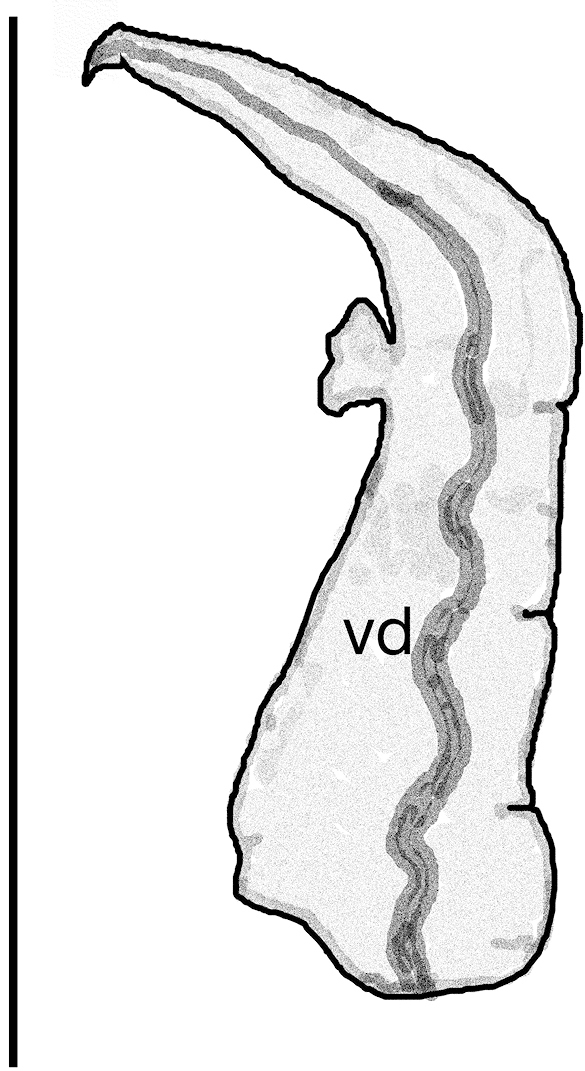
Penis of *Kerkia briani*. Abbreviation: vd – vas deferens.Scale bar: 1 mm.

Figures 4 , 5 , 6A, B , 7

####### Holotype.

Ethanol-fixed specimen (Fig. 4), spring Polički Studenac Vrelo (Crkvina), adjacent to the Trebišnjica River (42°42'46.4"N, 18°21'54.5"E), close to Trebinje (Bosnia and Herzegovina interstitially in the gravel 50 cm below the bottom of the spring. It is deposited in the Museum of Natural History of the University of Wroclaw, Poland, signature: MNHW-1350.

####### Paratypes.

Twelve paratypes, ethanol-fixed, in the collection of the Department of Malacology of Jagiellonian University.

####### Diagnosis.

Shell minute, nearly planispiral, distinguished from *K. kusceri* by its lower aperture of the shell and smaller non-glandular outgrowth on the left side of the penis, and from *K. jadertina* and *K. kareli* by its higher aperture of the shell and bigger the non-glandular outgrowth on the left side of the penis.

####### Description.

***Shell*** (Fig. 4) up to 0.77 mm high and 1.39 mm broad, nearly planispiral, whitish, translucent, thin-walled, consisted of approximately five whorls, growing rapidly and separated by moderately deep suture. Spire low and flat, body whorl large. Aperture prosocline, nearly circular in shape, peristome complete and thin, somewhat swollen, in contact with the wall of the body whorl; umbilicus wide, with the earlier whorls visible inside. Shell surface smooth, growth lines hardly visible.

***Measurements*** of holotype, sequenced, and illustrated shells: see Table 2. Shell variability slight (Fig. 5).

**Table 2.** Shell measurements (in mm) of *Kerkia briani*. For explanation of the symbols A–H, see Fig. 3.

	A	B	C	D	E	F	G	H
holotype	0.77	1.39	0.87	0.62	0.60	1.09	0.41	0.97
2D44	0.72	1.12	0.73	0.54	0.55	0.93	0.37	0.75
2F59	0.73	1.26	0.82	0.54	0.55	0.95	0.41	0.80
2F62	0.72	1.35	0.86	0.57	0.57	1.03	0.36	0.73
2F70	0.67	1.12	0.72	0.52	0.48	0.97	0.40	0.72
2F71	0.75	1.37	0.85	0.46	0.60	1.02	0.41	0.84
*M*	0.73	1.27	0.81	0.54	0.56	1.00	0.39	0.80
SD	0.034	0.123	0.067	0.053	0.044	0.059	0.023	0.094
Min	0.67	1.12	0.72	0.46	0.48	0.93	0.36	0.72
Max	0.77	1.39	0.87	0.62	0.60	1.09	0.41	0.97

***Soft parts morphology and anatomy.*** Body white, without pigment, with no eyes. The ctenidium with twelve short lamellae, osphradium short and broad. Rectum forming characteristic broad loop (Fig. 6A). The female reproductive organs (Fig. 6A, B) with a long, moderately broad loop of renal oviduct and relatively big spherical bursa copulatrix (Fig. 6A) with a long bent duct (Fig. 6B), and one distal receptaculum seminis, long and worm-shaped. The penis (Fig. 7) elongated triangular, with a rather sharp tip and small non-glandular outgrowth on its left side, the vas deferens inside running in zigzags.

####### Derivatio nominis.

The specific epithet *briani* refers to our friend Brian Lewarne, Honorary Science Officer of The Devon Karst Research Society, and the Director for “Proteus Project in the Trebišnjica River Basin”, deeply devoted to the protection of *Proteus* as well as the study and protection of the subterranean habitats in Bosnia and Herzegovina.

####### Distribution and habitat.

Known from the type locality only.

### Molecular distinctness and relationships of *Kerkia briani*

**Figure 8.**
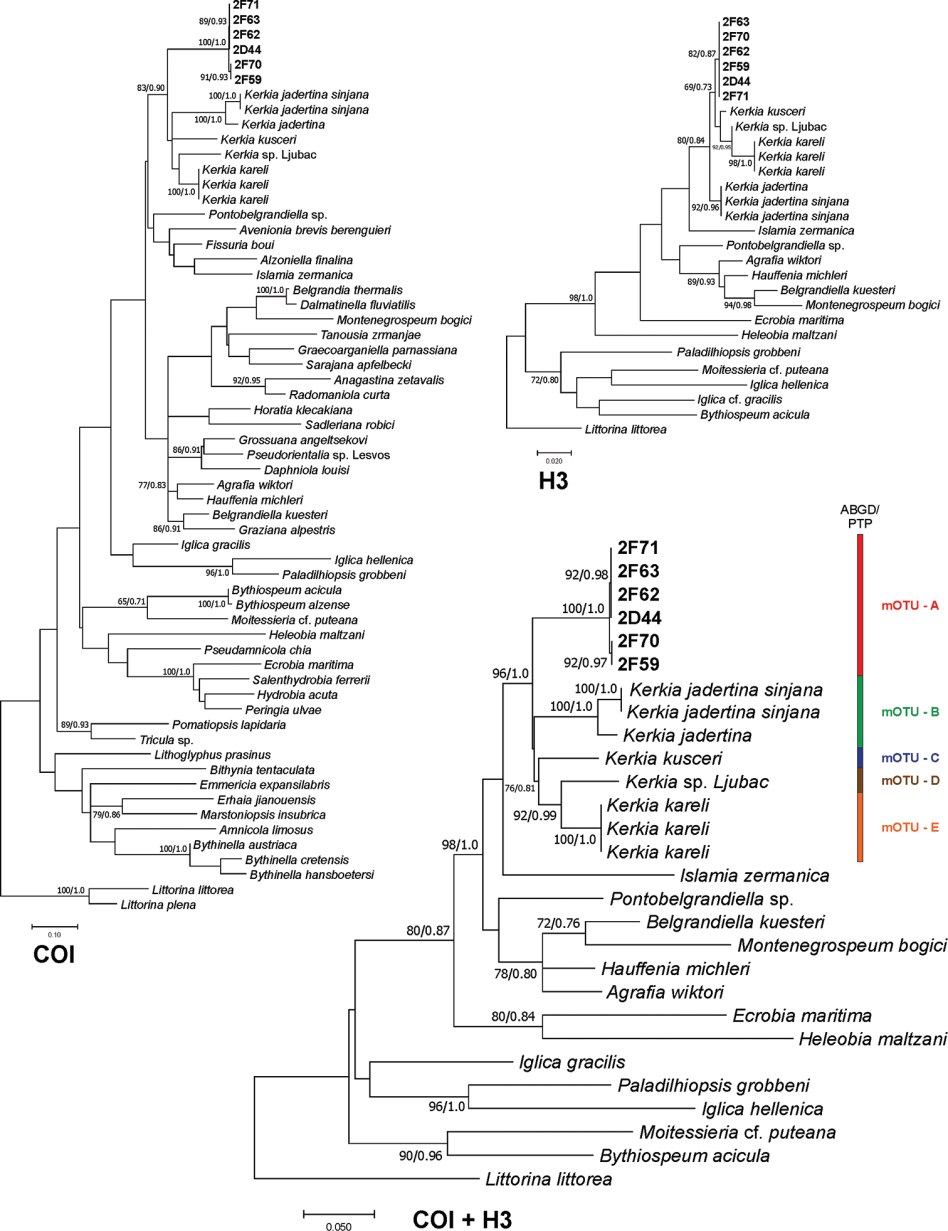
Maximum Likelihood tree inferred from mitochondrial COI. Bootstrap supports above 60% with corresponding Bayesian probabilities are given.

We obtained six new sequences of COI (479 bp, GenBank Accession Numbers MT780191–MT780196), and six new sequences of H3 (309 bp, GenBank Accession Numbers MT786730–MT786735). The tests by Xia et al. (2003) for COI and H3 revealed no saturation. Phylograms were constructed for COI, H3 and for combined COI-H3 dataset. In all analyses, the topologies of the resulting phylograms were identical in both the ML and BI. The ABGD and PTP approaches gave the same results (Fig. 8).

The sequences of the *Kerkia briani* formed distinct clade on COI, H3 as well as combined phylograms (Fig. 8). At the same time all *Kerkia* sequences formed distinct linage with five different species. The p-distance of *Kerkia briani* with other *Kerkia* species varied from 0.123 to 0.146 for COI and from 0.007 to 0.023 for H3 (Table 2). The sister clade of *Kerkia* (bootstrap support 98%) were *Islamia* Radoman, 1973, *Pontobelgrandiella* Radoman, 1978, *Belgrandiella* Wagner, 1927, *Montenegrospeum* Pešić & Glöer, 2013, *Hauffenia* Pollonera, 1898, and *Agrafia* Szarowska & Falniowski, 2011 (Fig. 8, the tree for concatenated COI and H3 sequences).

**Table 3.** p-distances between *Kerkia* mOTUs for the COI (below diagonal) and H3 genes.

	mOTU – A	mOTU – B	mOTU – C	mOTU – D	mOTU – E
mOTU – A	–	0.010	0.007	0.010	0.023
mOTU – B	0.135	–	0.017	0.020	0.033
mOTU – C	0.126	0.124	–	0.010	0.023
mOTU – D	0.146	0.138	0.124	–	0.013
mOTU – E	0.123	0.110	0.095	0.093	–

## Discussion

Following the terminology of Hershler and Ponder (1998), the habitus of the shell of *Kerkia* is depressed valvatiform (trochiform) or just planispiral. However, the latter term should not be used, since there is no planispiral shell in any recent gastropod (e.g., Falniowski 1993). The ctenidium, osphradium, and loop of oviduct are as in the other species of *Kerkia* (Bodon et al. 2001; Beran et al. 2014). The female reproductive organs are also typical of *Kerkia* (Radoman 1978, 1983; Bodon et al. 2001; Beran et al. 2014). The single receptaculum seminis is situated distally, in the position of rs_1_ after Radoman (1973, 1983). The penis is similar to that described and drawn by Radoman (1978, 1983), Bodon et al. (2001), and Beran et al. (2014), but the outgrowth of its left side in *K. briani* is smaller than in *K. kusceri*, but larger than that in *K. jadertina* and *K. kareli* (in the latter the outgrowth is nearly vestigial).

Falniowski (1987) demonstrated high variability of the shell, but also of the morphology and anatomy of the soft parts in the Truncatelloidea. In the latter, miniaturisation is one more a source of slight morphological diversity, decreasing the number of possible taxonomically useful characters (Falniowski 2018); in this regard, Szarowska and Falniowski (2008) stressed the narrow limits of morphology-based taxonomy within the Truncatelloidea. On the other hand, Szarowska (2006) demonstrated that such simple structures like the outgrowths on the penis and bursae/receptacula in the female reproductive organs are surprisingly evolutionary stable in position, although not in size and shape, whose variability – physiologically, ontogenetically, and artifactually (as a result of fixation of the snails) based – is striking. Moreover, problems can increase with taxa living in habitats of limited accessibility (such as caves and/or interstitial habitats) for which molecular studies often reveal numerous species but only a few or single living specimens of each species could be found. Thus, the anatomy is basic in distinction of the families and even genera, but the stable and reliable differences between congeneric species are hardly observable. However, the molecular distinctness of *Kerkia briani* is clear.

Finally, it has to be pointed out that *K. briani* inhabits the southernmost locality of *Kerkia*, expanding the range of the genus ca. 190 km ESE.

## Supplementary Material

XML Treatment for Kerkia briani
